# COL10A1 Facilitates Prostate Cancer Progression by Interacting With INHBA to Activate the PI3K/AKT Pathway

**DOI:** 10.1111/jcmm.70249

**Published:** 2024-12-10

**Authors:** Kun Jiang, Li‐zhe Xu, Fan Cheng, Jin‐zhuo Ning

**Affiliations:** ^1^ Department of Urology Renmin Hospital of Wuhan University Wuhan Hubei P.R. China

**Keywords:** COL10A1, INHBA, oncogene, PI3K/AKT pathway, prostate cancer

## Abstract

Prostate cancer (PCa) constitutes a highly common and lethal disease that impacts males globally. However, the specific molecular pathways responsible for its development are still unknown. Therefore, revealing the molecular regulators that contributed to the progression of PCa is pivotal for developing unique management strategies. Through comprehensive bioinformatics analysis of multiple public gene databases, we thoroughly investigated COL10A1 expression level, clinical significance, co‐expressed genes and signalling pathways in PCa. COL10A1 and INHBA expression level was assessed in clinical PCa specimens using RT‐qPCR, Western blotting and immunohistochemistry. A combination of experimental techniques, including CCK‐8 assay, colony formation, flow cytometry, Transwell, wound‐healing, immunoprecipitation assays and rescue study, was utilised to examine the fundamental molecular pathways of COL10A1's action across PCa. The COL10A1 expression was significantly elevated in PCa, and its upregulation has been connected with tumour aggressiveness and a weak predictive outcome in subjects. The current investigation revealed that regulation of COL10A1 expression, either by upregulation or downregulation, resulted in sequential augmentation or suppression of PCa cell progression, migration and invasion. Mechanistically, COL10A1 was manifested to directly interact with INHBA and facilitate PI3K and AKT phosphorylation pathways within PCa cells and mouse models. The results of our study offer new perspectives on the tumorigenic role of COL10A1 in PCa and its interactions with INHBA may play important roles in PCa progression.

**Abbreviations**
COL10A1: collagen type X alpha 1 chain
GEO: Gene expression omnibus
GO: Gene ontology
GSEA: gene set enrichment analysis
INHBA: inhibin subunit beta A
KEGG: Kyoto encyclopaedia of genes and genomes
PCa: Prostate cancer
PFI: Progression‐free interval
TCGA: The cancer genome atlas database

## 1 Introduction

Prostate cancer (PCa) represents a highly common malignancy in males and is recognised as the second‐most significant contributor to cancer‐related death in the male population worldwide, with an increasing incidence annually [1]. In 2022, it is projected that there will be more than 268,490 new PCa cases and 34,500 fatalities in the United States, representing approximately 27% of all cancer diagnoses in men [2]. Localised PCa can be treated through various options, including surgical removal of the prostate and radiotherapy. Patients with low‐risk or intermediate‐risk experienced PCa exhibit a promising prognosis, with a 10‐year overall survival rate of 99% when prompt identification and treatment are implemented [3, 4]. Despite the current advancements in clinical interventions for PCa, treating advanced‐stage PCa remains a significant therapeutic challenge [5]. Unfortunately, a subset of individuals diagnosed with primary PCa and subjected to initial therapeutic interventions will ultimately manifest metastatic PCa, a condition presently without any curative therapy options [6]. After the onset of metastases, the 5‐year survival rate for PCa experiences a notable decrease, reaching roughly 30% [7]. The development of PCa involves an extensive and multifaceted process, marked by intricate interactions amongst several determinants such as genetic factors, environmental influences and behaviours [8]. Despite the considerable clinical and experimental research undertaken in recent years, the specific molecular pathways underlying the development of PCa remain elusive. Therefore, identifying key molecular modulators involved in disease development would significantly bring about the advancement of novel management options for treating PCa individuals.

The COL10A1 gene, a collagen family member, codes for the alpha chain of collagen type X. Hypertrophic chondrocytes express this collagen gene during endochondral ossification [9]. In addition to its fundamental role in providing structural support, COL10A1 also has a crucial involvement in mediating cell–cell interactions. Elevated COL10A1 expression levels have consistently been observed across various malignant tumour types (including colon, lung, oesophageal, gastric, breast and bladder cancer) and are strongly linked to tumour progression, invasion, metastasis and angiogenesis [10, 11, 12]. The genes COL10A1 and the immune microenvironment have the potential to function as predictive indicators for neoadjuvant therapy in breast cancer [13]. Moreover, research conducted in vivo and in vitro has manifested that COL10A1 increases gastric cancer invasion and metastasis by triggering a process called epithelial‐mesenchymal transition (EMT) and activating the TGF‐β pathway [14]. However, the specific biological involvement and the molecular mechanisms underpinning COL10A1 function in progressing PCa are still not well understood.

The present investigation utilised gain‐ and loss‐of‐function methodologies both in vivo and in vitro to ascertain the potential involvement of COL10A1 as an oncogene in PCa progression while also exploring the underlying mechanisms driving these effects.

## 2 Materials and Methods

### 2.1 Bioinformatic Analysis

The sequencing counts and clinical information were acquired from The Cancer Genome Atlas (TCGA) data set (https://portal.gdc.cancer.gov/). The data was retrieved as fragments per kilobase per million (FPKM) at level 3 of HTSeq. The COL10A1 levels were estimated in 33 types of human malignancies, 501 cases of PCa and 52 non‐cancerous tissues employing data from TCGA. The data set pertaining to mRNA expression profile GSE38241 [15] was obtained from the GEO database (http://www.ncbi.nlm.nih.gov/geo) for analysis. The progression‐free interval (PFI) was assessed employing the “survival” and “survminer” R software, utilising Cox regression analysis. The pROC software in R has been utilised to construct the ROC curve for diagnostic applications. A correlation study of COL10A1 with T, N stages, Gleason score and age was conducted employing the “stats” and “car” packages in R. The “cor.test” utility in R was employed to assess the correlation measurements, utilising the Spearman method. The “clusterProfiler” package was utilised to perform enrichment analyses, encompassing Kyoto Encyclopedia of Genes and Genomes (KEGG), Gene Ontology (GO) and Gene set enrichment analysis (GSEA). The gene sets utilised in the GSEA were obtained by accessing the Molecular Signatures Database (MsigDB) hallmark and C2‐curated data sets.

### 2.2 Clinical Tissues

Between the years 2019 and 2021, a collection of 38 PCa tissues and their equivalent non‐malignant specimens were procured from the Department of Urology at Renmin Hospital of Wuhan University. The normal control tissue was carefully selected to be as distant from the tumour as possible. The experiment received approval from the Clinical Research Ethics Committee of Renmin Hospital of Wuhan University, with all participating patients signing informed consent. The excised tissues were promptly collected and cryopreserved in liquid nitrogen. All specimens underwent independent and blinded evaluation by two expert pathologists. The clinicopathological characteristics of the 38 cases diagnosed with PCa were thoroughly documented and recorded in Table 1.

**TABLE 1:** The clinicopathological features of 38 patients with prostate cancer and their correlations with the expression of COL10A1.

Characteristics	Cases (*n* = 38)	COL10A1 expression		*p*
		Low (*n* = 16)	High (*n* = 22)	
Age				
≤ 60	14	10	4	0.0077*
> 60	24	6	18	
T stage				
T1–2	9	7	2	0.0211*
T3–4	29	9	20	
N stage				
N0	22	13	9	0.0202*
N1	16	3	13	
Gleason score				
6 and 7	11	8	3	0.028*
8–10	27	8	19	

* *p* < 0.05 considered statistically significant.

### 2.3 Cell Lines and Culture

The DU145, LNCaP, PC3, HEK293T and RWPE‐1 cell lines were acquired from the Cell Bank of the Chinese Academy of Sciences (Shanghai, China). The RWPE‐1 cells were cultivated in K‐SFM media with bovine pituitary extract and human epidermal growth factor at 37°C and 5% CO_2_ concentration. RPMI‐1640 media was used to cultivate PCa cells, which were enriched with 10% foetal bovine serum (FBS) and 1% penicillin. Dulbecco's modified Eagle medium (DMEM) was employed to cultivate the HEK293T cells.

### 2.4 Cell Transfection

Flag‐tagged COL10A1 overexpression plasmids, HA‐tagged INHBA overexpression plasmids, shRNA against COL10A1, shRNA against INHBA and the corresponding control with nonsense sequences were custom‐designed and created via GeneChem (Shanghai, China). The transfection procedure has been conducted in accordance with established protocols. The selection of cell lines with stable gene expression was performed using puromycin, and the transfection efficacy was subsequently validated through real‐time quantitative polymerase chain reaction (RT‐qPCR) analysis.

### 2.5 RNA Extraction and RT‐qPCR

The total RNA was isolated from prostate specimens and cultivated PCa cells employing TRIzol reagent (Invitrogen, USA) according to the manufacturer's instructions. The total RNA was subsequently converted into cDNA through reverse transcription utilising the PrimeScript RT reagent Kit (Takara, China). The detection of PCR products was accomplished through the SYBR Green PCR Master Mix (Roche, Switzerland). To achieve precise measurements, the relative mRNA expression levels of the target genes were normalised by aligning them with the average GADPH expression level. The RT‐qPCR analysis was carried out employing a Roche LightCycler 480 instrument, following the stated methodology. The primer sequences were as follows: COL10A1 (forward: 5'‐ACCCAAGGACTGGAATCTTTAC‐3', reverse: 5'‐GCCATTCTTATACAGGCCTACC‐3'); INHBA (forward: 5'‐TCTGCAGTAGTGTGGACTAGAA‐3', reverse: 5'‐CCTGGGTAATTGGGTAGGAAAG‐3'); GAPDH (forward: 5'‐CCTGCACCACCAACTGCTTA‐3', reverse: 5'‐TCTTCTGGGTGGCAGTGATG‐3').

### 2.6 Immunohistochemistry Staining

The human PCa tissues were subjected to fixation using a 4% paraformaldehyde solution, followed by drying, embedding in paraffin, and sectioning into 4‐μm thick slices. Afterwards, the slides were kept at 4°C overnight and treated with an anti‐COL10A1 antibody (1:1000, ab49945, Abcam, China). After being washed via PBS, the sections underwent incubation using secondary antibodies for 30 min at normal temperature. Then, they were further incubated with streptavidin‐horseradish peroxidase at 37°C for 20 min. Subsequently, the slides underwent incubation employing 3, 3‐diaminobenzidine tetrahydrochloride as the chromogen, followed by counterstaining with haematoxylin and bluing reagent. The observation of the slides was conducted utilising the Olympus B × 50 light microscope (Olympus, Japan).

### 2.7 Western Blotting (WB)

The total protein extraction was conducted using RIPA buffer (Beyotime, China), quantifying its concentration via a BCA protein assay reagent (Beyotime, China). The proteins were separated employing 10% SDS polyacrylamide gels (Solarbio, China) and subsequently deposited onto PVDF membranes (Bio‐Rad, USA). After the application of a 5% non‐fat milk solution to inhibit non‐specific binding, the blots were then subjected to overnight incubation at 4°C employing primary antibodies specific to COL10A1 (1:1000, ab182563), INHBA (1:1000, ab128958), PI3K (1:2000, ab140307), p‐PI3K (1:500, ab182651), AKT (1:1000, ab8933), p‐AKT (1:1000, ab38449), and GAPDH (1:1000, ab8245), which were obtained from Abcam (Shanghai, China). Following incubation with the secondary antibody, the signals were identified employing an enhanced chemiluminescence (ECL) detection system (Bio‐Rad, USA).

### 2.8 Cell Counting Kit 8 (CCK‐8) Assay

The cellular proliferative viability was evaluated by employing the CCK‐8 Assay Kit (Biosharp, China). The cells underwent inoculation in 96 wells at 1000 cells per well, transfected for interval times of 0, 24, 48 and 72 h, and subsequently incubated with the CCK‐8 reagent at 37°C for 2 h. Using a microplate reader (Perkin‐Elmer, USA), the absorbance of each well was calculated.

### 2.9 Colony Formation Assay

The evaluation of cell viability was performed utilising a colony formation test. The PCa cells were cultivated in 6‐well plates, with each well containing a seeding number of 2000 cells. The cells underwent incubation at 37°C for a duration of 2 weeks in an incubator. The culture media was refilled at three separate intervals each week to ensure the maintenance of ideal growth conditions. After the specified incubation time, the observable cell colonies in each well were subjected to a 0.1% solution of crystal violet dye for 15 min, enabling enhanced visualisation and quantification of the colonies.

### 2.10 Cell Apoptosis Assay

The PC3 cells had two rounds of staining with the Annexin V‐FITC apoptosis detection kit (Beyotime, China) following the manufacturer's recommendations and incubated at normal temperature for 30 min. The proportion of apoptotic cells was ascertained utilising a flow cytometer (Beckman Coulter, USA).

### 2.11 Transwell (TW) Assay

The TW assays were classified into two separate groups: TW migration and invasion. The evaluation of cellular migration and invasion was performed employing 24‐well TW chambers that were fitted with 8‐μm pore membranes (Corning LifeSciences, USA). The invasion tests have been conducted utilising Matrigel‐coated TW chambers (Corning LifeSciences, USA). The PCa cells have been cultivated with a serum‐free media for a duration of 24 h. Afterwards, they were transferred to the upper chamber of the TW insert, whereas the lower chamber was supplemented with a medium comprising 10% FBS to serve as a chemoattractant for facilitating cellular migration and incubated at 37°C for 48 h, followed by gentle rinsing with PBS and fixation using 100% methanol at a lower temperature for 15 min. After that, the cells were subjected to staining using crystal violet solution (0.05%) at room temperature, and five random fields were selected for subsequent analysis. Matrigel‐free TW chambers were employed to evaluate cellular migration. The cell migration assay procedures strongly resembled that of the invasion experiment.

### 2.12 Wound‐Healing Assay

The PC3 cells have been transfected and subsequently cultivated in six‐well plates until they achieved 80%–90% confluence. Using 200 μL plastic pipette tips, the wounds were created by gently abrading the cell layers. Subsequently, the plates were cultured in serum‐free media following two or three washes with PBS in each well. The migratory distances of cells were measured and recorded using microscopic imaging (Olympus, Japan) at two specific time points: 0 and 24 h following the creation of a scratch.

### 2.13 Immunoprecipitation (IP) Assay

For coimmunoprecipitation (Co‐IP) experiments, plasmids were co‐transfected into HEK293T and PC3 cells. After a 24‐h incubation period, the cells were harvested using a cold IP lysis buffer enriched with phosphatase and protease suppressors. The cellular lysates were subjected to centrifugation, resulting in protein‐rich supernatant formation. This supernatant was then tested with IP employing protein G‐coated agarose beads. The resultant combination underwent incubation at 4°C overnight using specific antibodies targeting the desired tag. To conduct endogenous Co‐IP, the PC3 cells were lysed utilising an IP buffer, subjecting the lysates to IP using specific primary antibodies that were suitable for the objective analysis. Afterwards, the beads underwent extensive washing employing lysis solution, then resuspended in 2 × SDS loading buffer and boiled. Subsequently, the obtained specimens were subjected to SDS‐PAGE analysis in order to ascertain protein interactions.

### 2.14 Xenograft Tumour Model

The nude mice (4 weeks old), acquired from the Centre of Experimental Animals at Wuhan University Medicine College, were kept in a temperature‐controlled isolation facility and were given unrestricted availability of food and water. The animal care and experimental techniques followed the National Institutes of Health Guidelines for Laboratory Animal Use. For the nude mouse xenograft experiment, each nude mouse was subcutaneously administered 2 × 10^6^ PC3 cells suspended in 100 μL of PBS. The tumour size was evaluated on a weekly basis after injection using a vernier calliper, and the volumes of the tumours were determined by applying the upcoming formula: Volume = (Length × Width^2^)/2. Following 35 days, the mice were killed following ethical recommendations, and their tumours were then gathered for additional investigation.

### 2.15 Statistical Analysis

The statistical analyses have been conducted employing Prism 8.0 and SPSS 22.0 software. Bioinformatics analysis was conducted in R version 4.2.1. The mean ± standard deviation (SD) of the continuous data was calculated in at least three separate investigations. The student's *t*‐test was deployed to establish the statistical significance between two distinct groups, while one‐way analysis of variance (ANOVA) was employed to conduct comparisons containing more than two groups. The determination of statistical significance has been achieved based on a two‐sided *p* < 0.05.

## 3 Results

### 3.1 COL10A1 Is Upregulated in PCa and Might Function as an Accurate Indicator for Both Diagnosis and Prognosis

**FIGURE 1:**
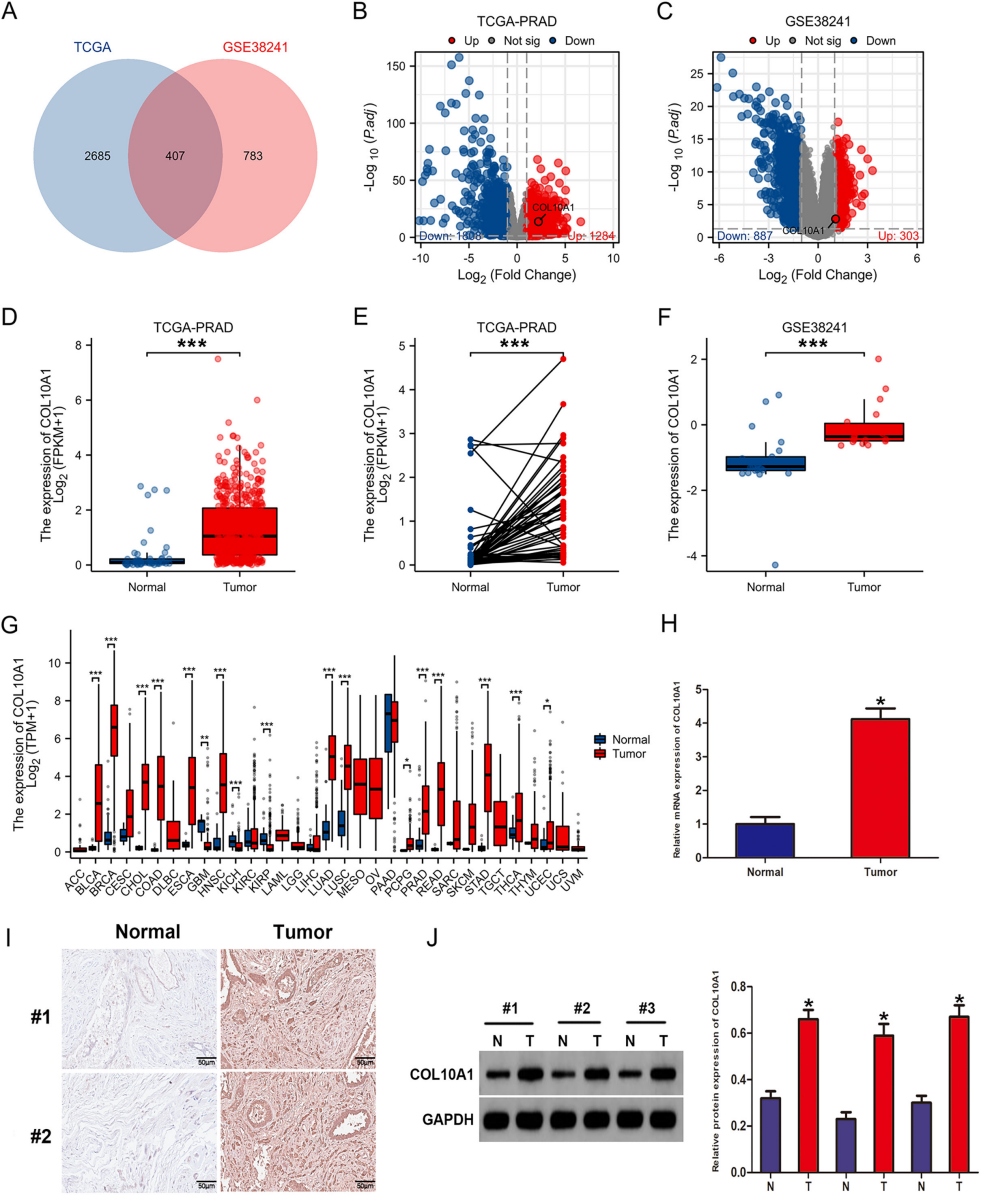
COL10A1 is heightened in PCa. (A) Venn diagram: Intersection of the DEGs originated from TCGA‐PRAD and GSE38241. (B, C) The volcano plot: The whole range of gene expression alterations in both the TCGA‐PRAD and the GSE38241 data sets. (D, E) TCGA‐PRAD data set: PCa tumour tissues had an elevated COL10A1 expression contrasted with normal tissues. (F) GSE38241 data set: COL10A1 expression is heightened in PCa tumour tissues as opposed to normal tissues. (G) TCGA database: COL10A1 expression in multiple cancers. (H) RT‐qPCR: COL10A1 expression level in PCa and normal specimens. (I) Immunohistochemical staining images: COL10A1 expression in PCa tissues, along with corresponding normal tissues. (J) WB analysis: COL10A1 expression in PCa and normal tissues paired with PCa. *p < 0.05; **p < 0.01; ***p < 0.001.

**FIGURE 2:**
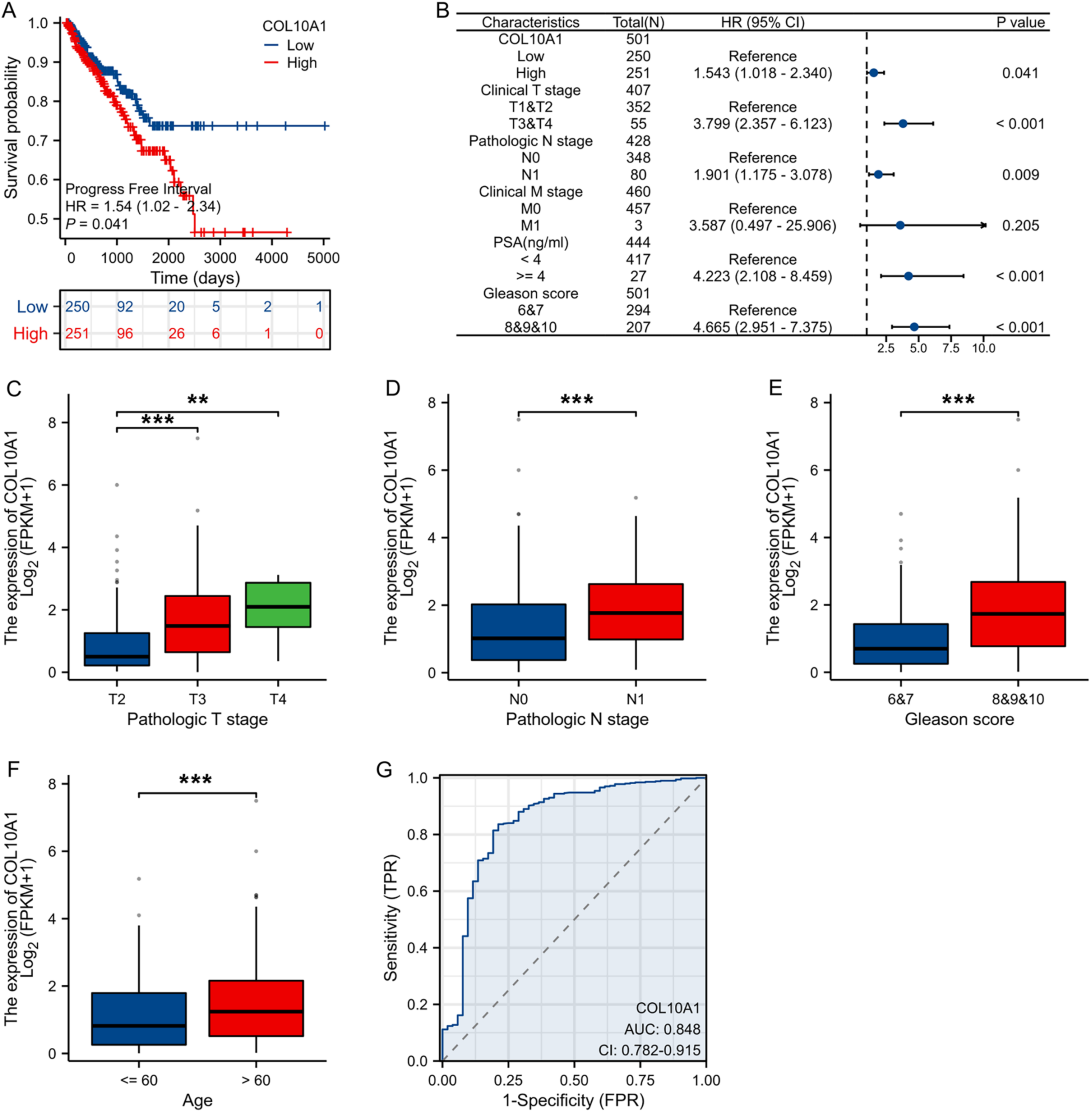
Comprehensive predictive, clinicopathological and diagnostic analysis of COL10A1 in PCa. (A) PFI curve of PCa individuals having low and high COL10A1 expression levels within TCGA database. (B) Univariate analyses of prognostic parameters in TCGA database PFI. (C–F) COL10A1 relative expression levels in TCGA‐PRAD data set with pathologic T/N stages, Gleason score and age. (G) ROC curve: Heightened COL10A1 expression specificity in both PCa and normal tissues. **p < 0.01; ***p < 0.001.

Gene expression profiles from the TCGA‐PRAD and the GSE38241 data sets were analysed to determine the molecular mechanisms linked to PCa. The determination of differentially expressed genes (DEGs) depended on a threshold of an adjusted p < 0.05 and |log2 FC| > 1. Based on the TCGA‐PRAD data set, the volcano plot indicated that amongst the 3092 DEGs, 1284 genes underwent overexpression, and 1808 genes underwent underexpression in PCa tissue samples compared to normal tissue samples (Figure 1B). The volcano plot revealed that a total of 1190 DEGs underwent screening (303 overexpressed and 887 underexpressed) from the GSE38241 data set (Figure 1C). Venn diagram demonstrating the intersection of the 407 DEGs originated from TCGA‐PRAD and GSE38241 (Figure 1A). COL10A1 was obtained from the intersection of the DEGs for further analysis. Using TCGA data, we next revealed that the COL10A1 expression levels were remarkably elevated within PCa tissue samples contrasted with normal tissue samples (Figure 1D,E). The GSE38241 data set revealed that COL10A1 was overexpressed within PCa tissue samples contrasted with normal tissue samples (Figure 1F). Furthermore, many malignancies manifested a significant rise in the amount of COL10A1 present when contrasted with corresponding healthy tissues (Figure 1G). Subsequently, we examined the expression of COL10A1 in PCa specimens. RT‐qPCR, immunohistochemical staining and WB manifested a significant rise within the COL10A1 expression levels across PCa tissues as opposed to normal tissue samples (Figure 1H–J). The Kaplan–Meier survival curves showcased that PCa patients having COL10A1 overexpression exhibit a weak progression‐free interval (PFI) contrasted to those having low COL10A1 expression (Figure 2A). In addition, our analysis manifested that the COL10A1 expression was a predictive predictor, regardless of other known prognostic variables, in the univariate Cox regression analyses. These factors encompassed T/N/M stages, PSA and Gleason score (Figure 2B). In addition, our examination of the TCGA database manifested significant connections between the COL10A1 expression and other clinical features, encompassing T, N stages, Gleason score and age (Figure 2C–F). The predictive accuracy of COL10A1 on PCa was assessed employing ROC analysis, yielding a projected AUC of 0.848 (Figure 2G). Our findings demonstrate that the expression of COL10A1 exhibits a statistically significant overexpression within PCa tissues and is correlated to the malignant progression of the disease.

### 3.2 COL10A1 Promotes PCa Cell Proliferation and Inhibits Apoptosis

**FIGURE 3:**
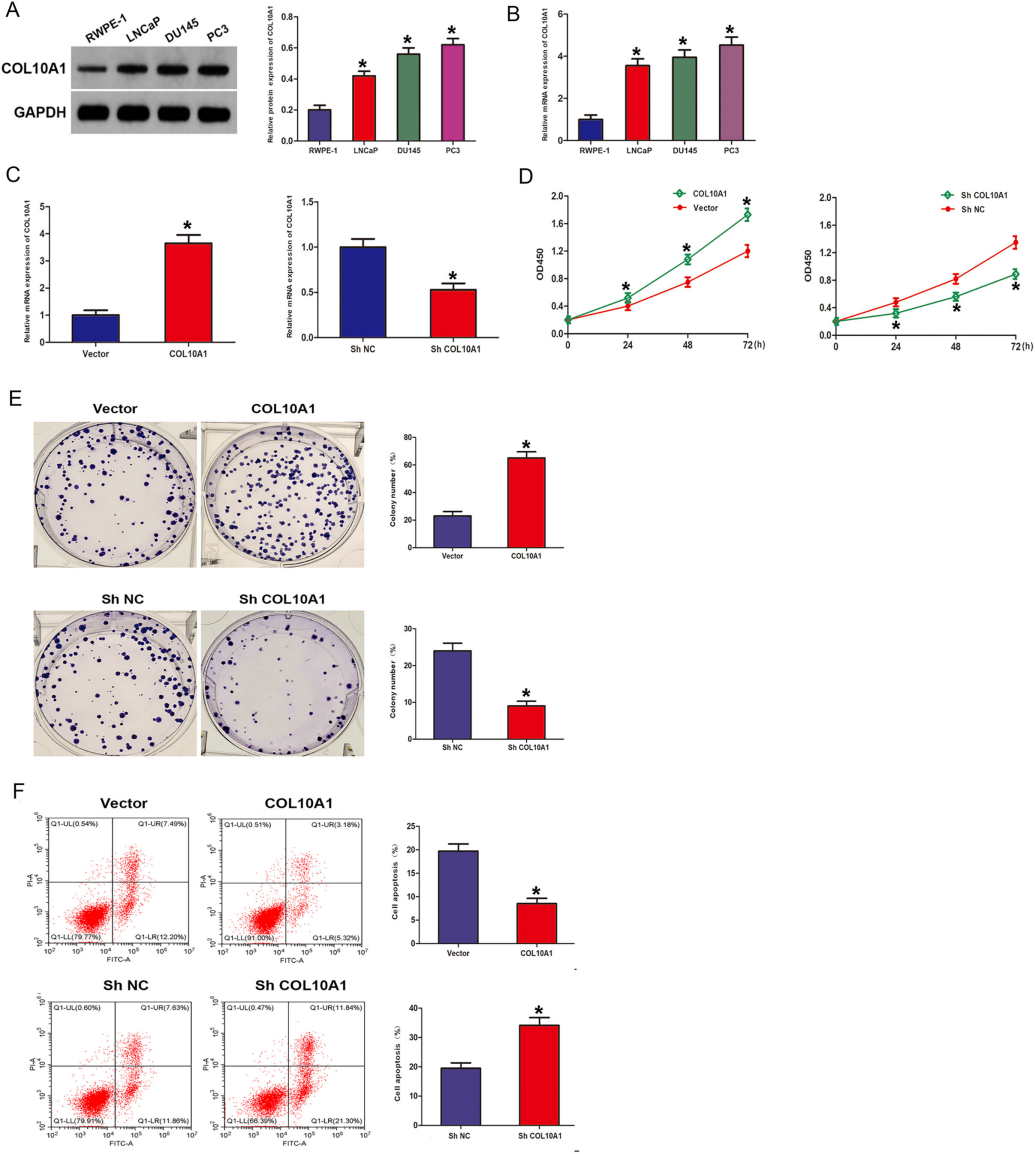
COL10A1 elevates PCa cell proliferation and hinders apoptosis In Vitro (A, B) WB and RT–qPCR: COL10A1 levels in the RWPE‐1, LNCaP, DU145, and PC3 cell lines. (C) RT‐qPCR: Validation of COL10A1 upregulation and downregulation effects. (D) CCK‐8: PC3 cell proliferation throughout 0 to 72 h. (E) Colony formation: PC3 cells proliferative capacity. (F) Flow cytometry: PC3 cell apoptosis rate. *p < 0.05.

Initially, we assessed the COL10A1 expression across multiple cell lines (LNCaP, DU145 and PC3) as well as in normal prostate epithelium (RWPE‐1) through RT‐qPCR and WB. All PCa cells, especially PC3 cells, showed a significant rise in the COL10A1 expression contrasted with RWPE‐1 cells (Figure 3A,B). The effectiveness of the COL10A1 overexpression and COL10A1 knockdown was verified utilising RT‐qPCR (Figure 3C). The COL10A1 implications on PCa cell proliferation were examined by employing CCK‐8 and colony formation assays, showcasing that PC3 cells overexpressing COL10A1 had significantly higher cell viability features contrasted with the vector group. Conversely, the sh‐COL10A1 group manifested a statistically significant hindrance in cell viability features as opposed to those found in the shNC group (Figure 3D). Similarly, our colony formation assay outcomes demonstrated that upregulation of COL10A1 exhibited statistically significant enhancement in the context of cell growth and proliferation, whereas downregulation of COL10A1 suppressed colony formation in vitro (Figure 3E). The flow cytometry outcomes manifested that COL10A1 upregulation significantly mitigated the number of apoptotic nuclei, while suppression of COL10A1 expression restored the apoptotic activity of the cells (Figure 3F). Collectively, this investigation findings revealed that COL10A1 performs a crucial function in inhibiting apoptosis and mediating the proliferation of PCa cells.

### 3.3 COL10A1 Enhances PCa Cell Migration and Invasion

**FIGURE 4:**
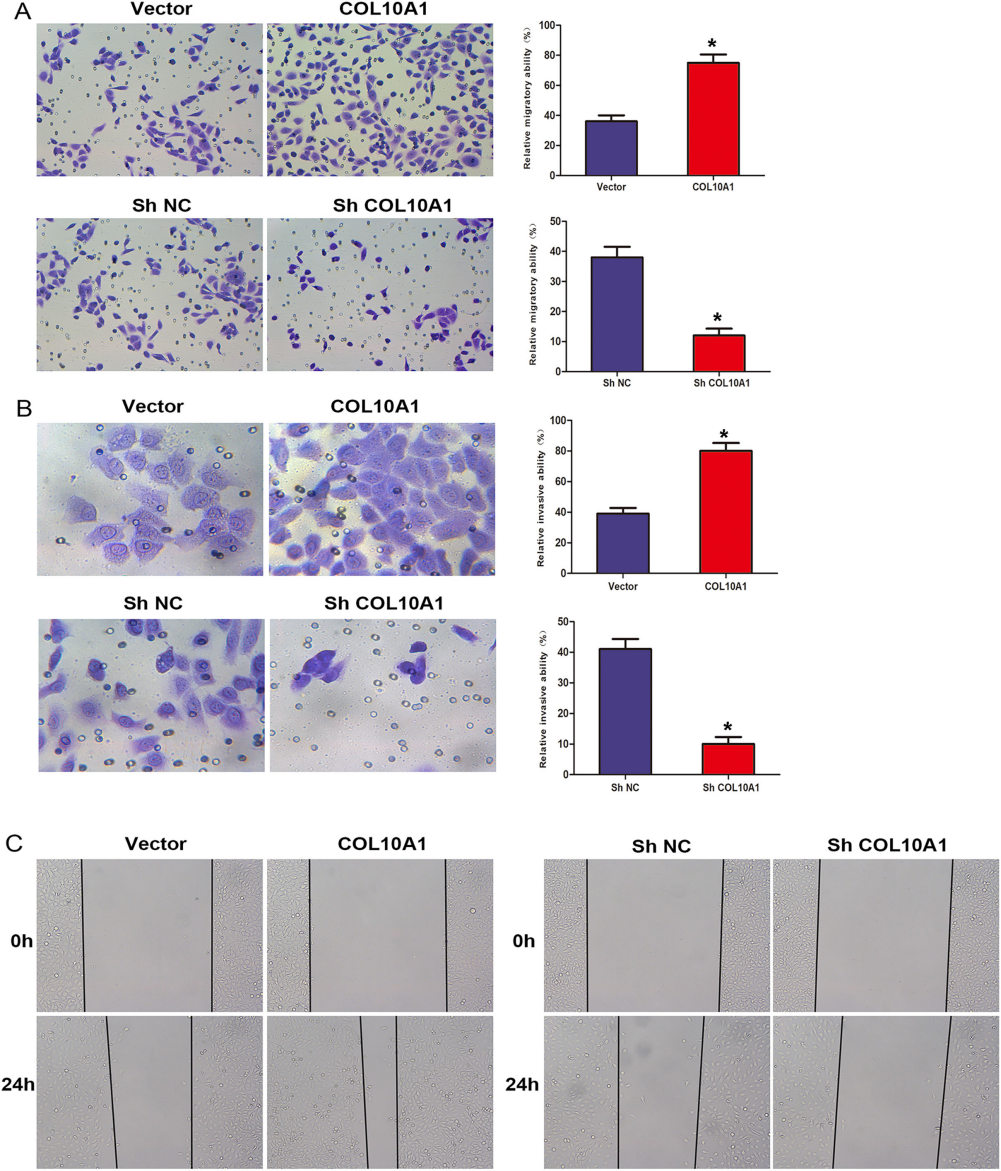
COL10A1 mediates the PCa cell migration and invasion. (A) TW migration assessment: PC3 cells metastasis capacity. (B) TW invasion assay: PC3 cells invasion capacity. (C) TW invasion experiments: PC3 cells invasion potential with Matrigel‐coated membranes. *p < 0.05.

To estimate the COL10A1 implications on the migratory and invasive features of PCa cells, we performed TW and wound‐healing tests. The outcomes obtained from the TW migration experiment manifested that the rise in COL10A1 expression significantly enhanced the PC3 cells migration, whereas a decline in COL10A1 expression resulted in a drop in migration (Figure 4A). Furthermore, our TW invasion tests showed that the elevated COL10A1 expression significantly enhanced the PC3 cell invasion, while the mitigated COL10A1 expression hindered invasion (Figure 4B).

Consistent with the TW assay results, our wound‐healing assays demonstrated that COL10A1 upregulation significantly improved PC3 cell migration, whereas COL10A1 downregulation significantly reduced cell migration (Figure 4C). Collectively, COL10A1 is crucial in enhancing PCa cell invasion and migration.

### 3.4 COL10A1 Co‐Expressed Genes and Functional Enrichment Analysis in TCGA‐ PRAD Patients

**FIGURE 5:**
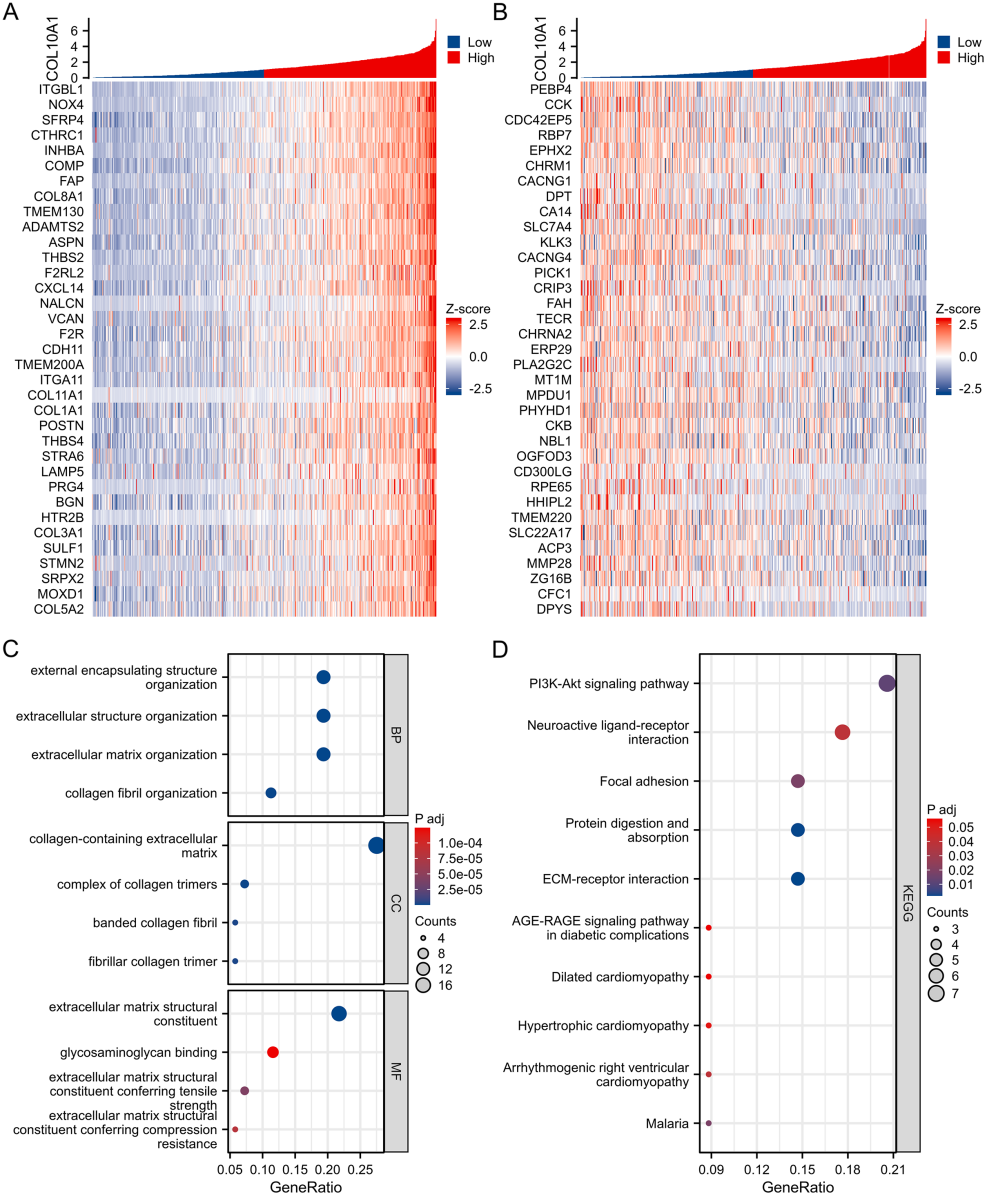
COL10A1 co‐expressed genes and functional enrichment analysis in TCGA‐PRAD patients. (A, B) Heatmap and (C, D) GO and KEGG pathway analyses: Top 35 genes positively and negatively linked to COL10A1 in PCa.

In order to enhance comprehension regarding the possible biological functions of COL10A1 in PCa, we carried out a co‐expression analysis utilising data from the TCGA database. The drawn heatmaps to visually display the top 35 genes demonstrated a significant positive connection with COL10A1 in PCa, as well as the top 35 genes that manifested a significant adverse connection with COL10A1 in PCa (Figure 5A,B). In order to obtain additional knowledge regarding the prospective functions and underlying biological mechanisms of the highest 35 genes that exhibit positive and negative correlations with COL10A1 in PCa, we conducted GO and KEGG pathways analyses. The GO investigation manifested that these genes had a significant enrichment in the collagen‐containing extracellular matrix and had a structural function in the extracellular matrix (Figure 5C). The KEGG pathway analysis revealed that the highest 35 genes emerging positive and negative connections with COL10A1 in PCa were primarily associated with enrichment in the PI3K/AKT signalling pathway (Figure 5D).

### 3.5 COL10A1 Regulates INHBA Expression in PCa Cells

**FIGURE 6:**
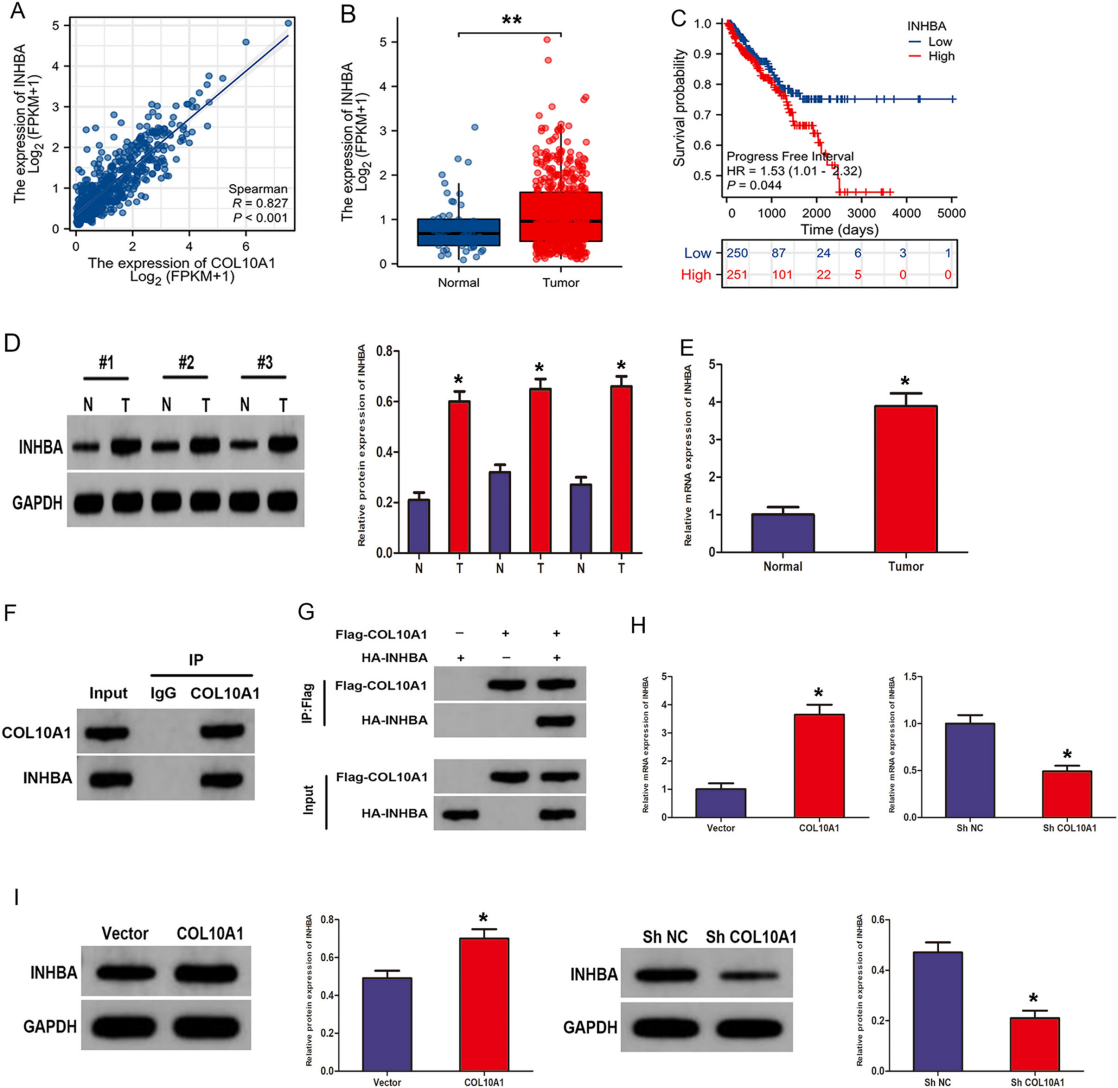
COL10A1 modulates the expression of INHBA within PCa cells. (A) TCGA‐PRAD data set: COL10A1 expression emerged as a significant positive link to INHBA expression. (B) TCGA‐PRAD data set: INHBA was overexpressed in PCa. (C) The PCa PFI curve in the TCGA database includes individuals with low and high INHBA expression. (D, E) WB and RT‐qPCR: INHBA differential expression levels in PCa and normal specimens. (F) Co‐IP: The interactions between endogenous COL10A1 and endogenous INHBA in PC3 cells. (G) Co‐IP: The interactions between exogenous COL10A1 and exogenous INHBA in HEK293T cells transfected with the indicated constructs. (H, I) RT‐qPCR and WB: INHBA expression in PC3 cells transfected with shCOL10A1 or COL10A1 overexpression vector. *p < 0.05; **p < 0.01.

To gain a deeper understanding of the molecular mechanism of COL10A1 in PCa, we performed co‐expression analysis utilising data from the TCGA database and STRING database. Significantly, our analysis revealed a potential correlation between COL10A1 and INHBA, prompting us to select INHBA for further investigation. An investigation was performed to examine the link between the COL10A1 and INHBA expression levels in TCGA‐PRAD patient data, manifesting a significant positive relation between both genes in PCa (Figure 6A). Based on the TCGA data, the PCa tissues experienced a significantly overexpressed INHBA as opposed to normal tissues (Figure 6B). As the Kaplan–Meier survival curves showed, PCa individuals exhibiting greater INHBA levels revealed worse PFI (Figure 6C). WB and RT‐qPCR manifested a significantly higher INHBA expression across PCa tissues than in normal tissues (Figure 6D,E). Consequently, we further verify whether COL10A1 directly interacts with INHBA. The connection between COL10A1 and INHBA was verified by employing endogenous (Figure 6F) and exogenous (Figure 6G) Co‐IP tests in PC3 and HEK293T cells, respectively. Moreover, RT‐qPCR and WB indicated that upregulation of COL10A1 significantly elevated the expression level of INHBA, while knockdown of COL10A1 down‐regulated it (Figure 6H,I).

### 3.6 INHBA Promotes COL10A1 to Mediate the Malignant Behaviour of PCa Cells

**FIGURE 7:**
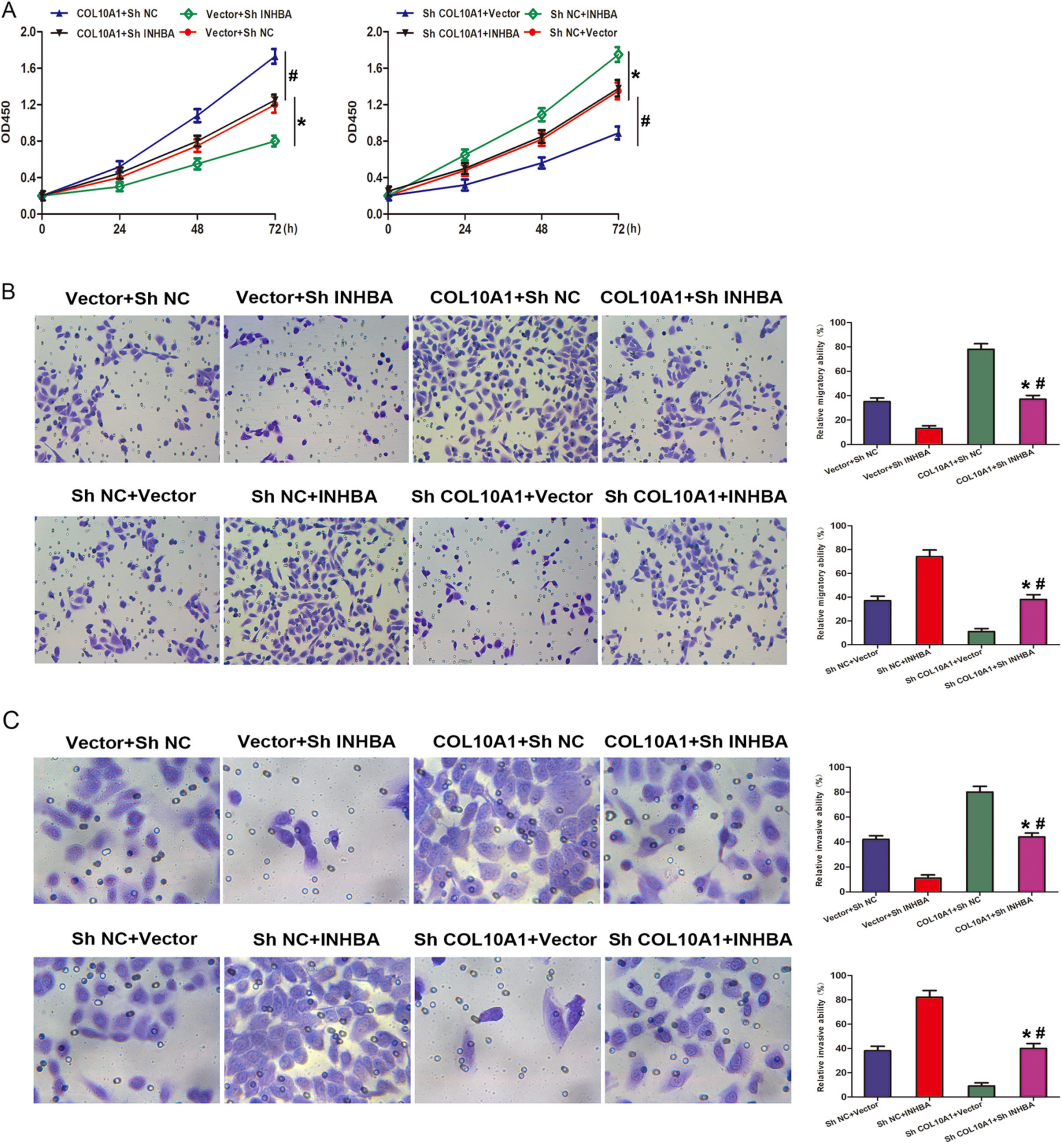
COL10A1 promotes PCa cell progression, migration and invasion through interacting with INHBA. PC3 cells transfected with the COL10A1 upregulation vector and/or shINHBA and with the INHBA upregulation vector and/or shCOL10A1. (A) CCK‐8 assay: PC3 cell proliferation from 0 to 72 h post‐transfection. (B) TW migration assay: Metastasis capacity of PC3 cells after transfection. (C) TW invasion experiments: Invasion potential of PC3 cells with Matrigel‐coated membranes. *p < 0.05; ^#^ *p* < 0.05.

After establishing the regulatory association between COL10A1 and INHBA, we proceeded to conduct further investigation into the involvement of the COL10A1‐INHBA axis in the biological functioning of PCa. The findings from the CCK‐8 assay showed that the COL10A1 upregulation led to an increase in cell proliferation, which was afterwards reversed by the downregulation of INHBA. Conversely, the downregulation of COL10A1 resulted in a hindrance in cell proliferation, which was subsequently reversed by the INHBA upregulation (Figure 7A). Similarly, the findings obtained from TW assays revealed that silencing INHBA restored the increase in cell migration and cell invasion stimulated via COL10A1 upregulation, whereas upregulation of INHBA restored the hindrance in cell migration and cell invasion induced through suppressing COL10A1 (Figure 7B,C). These data show that COL10A1 enhances the proliferative, migrating and invading capabilities of PCa cells by boosting the INHBA expression.

### 3.7 COL10A1‐INHBA Axis Stimulates the PI3K/AKT Pathway Across PCa Cells

**FIGURE 8:**
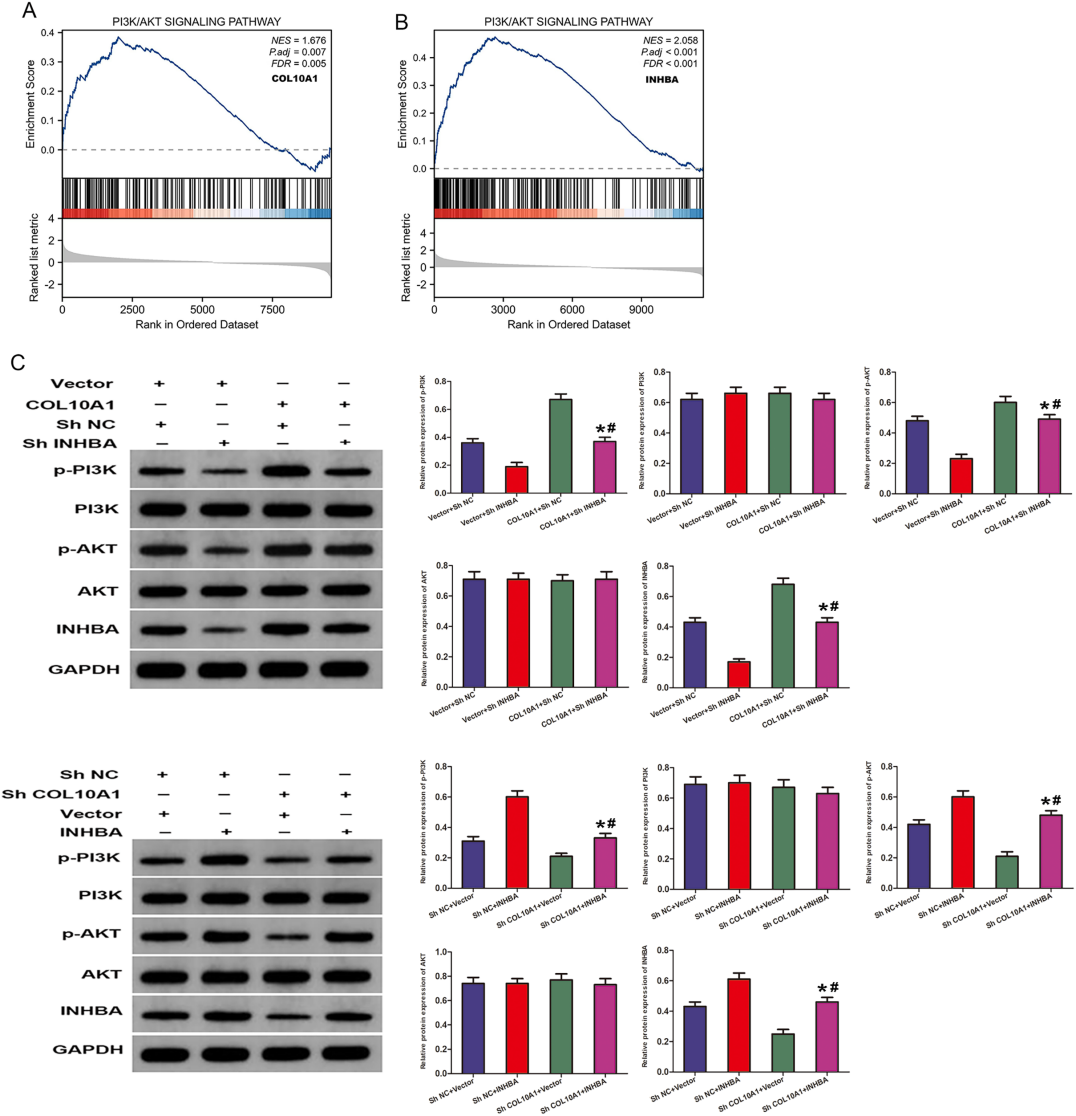
COL10A1‐INHBA axis activates PI3K/AKT pathway in PCa cells. (A, B) GSEA: PI3K/AKT pathway enriched in the high COL10A1 and INHBA expression groups. (C) WB analysis: P‐PI3K, PI3K, p‐AKT and AKT protein expression levels in the PC3 cells infected with shCOL10A1 and/or INHBA overexpression vector. *p < 0.05; ^#^ *p* < 0.05.

The TCGA data analysis utilising GSEA suggested a significant enrichment in the PI3K/AKT pathway in PCa specimens that had greater COL10A1 or INHBA expression levels (Figure 8A,B). A WB study was applied to investigate whether the COL10A1‐INHBA axis controls the PI3K/AKT pathway by examining the downstream gene expression levels in this pathway. The WB analysis demonstrated that the INHBA inhibition resulted in the restoration of the greater levels of PI3K and AKT phosphorylation that were produced by the COL10A1 overexpression. In contrast, the excessive production of INHBA reversed the decline in p‐PI3K and p‐AKT expression stimulated via the COL10A1 suppression (Figure 8C). Therefore, the aforementioned findings indicate that COL10A1 stimulates the PI3K/AKT pathway via modulated INHBA expression in PCa cells.

### 3.8 Inhibition of COL10A1 Suppresses PCa Development Through Suppressing the PI3K/AKT Pathway and INHBA In Vivo

**FIGURE 9:**
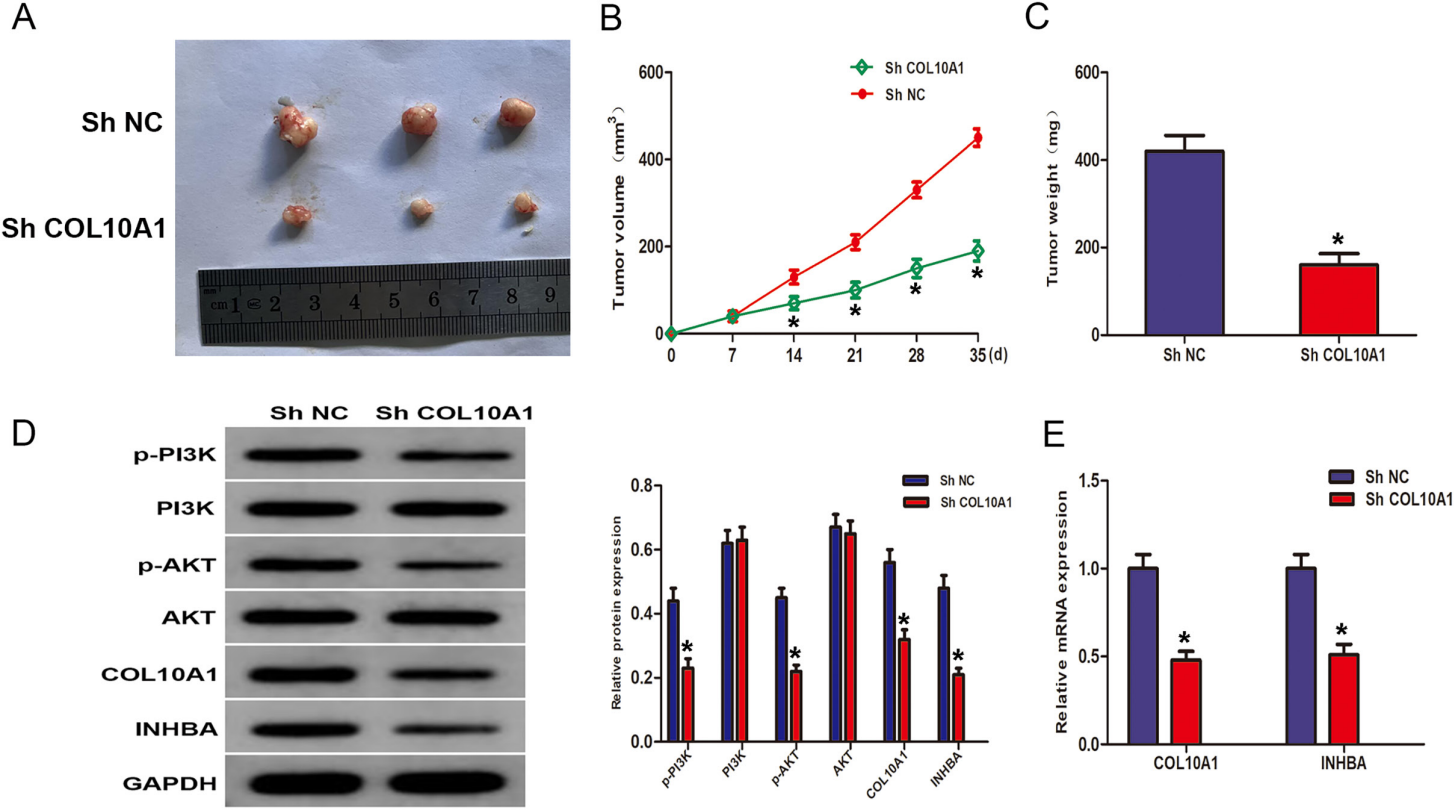
COL10A1 promotes tumorigenic behaviour in PCa cells by activation of PI3K/AKT In Vivo. (A) The shNC or shCOL10A1‐transfected PC3 cells were the source of the subcutaneous xenograft tumours. (B) Xenograft tumour volume in the shNC and shCOL10A1 cohorts. (C) Xenograft tumours weight in shNC and shCOL10A1 groups. (D) WB: PI3K/AKT‐related proteins, COL10A1 and INHBA in xenograft tumours in shNC and shCOL10A1 groups. (E) RT‐qPCR: COL10A1 and INHBA in xenograft tumours in shNC group and shCOL10A1. *p < 0.05.

To conduct a more comprehensive examination of the impact of COL10A1 in vivo, the PC3 cells transfected with shCOL10A1 lentivirus into mice underwent subcutaneous injection, and then they were monitored for changes in tumour volume every 7 days. The findings of our study indicate a significant reduction in both volume and weight of subcutaneous tumours in the shCOL10A1 group as opposed to the shNC group (Figure 9A–C). WB analysis manifested that the shCOL10A1 group possessed a significant hindrance in the phosphorylation of PI3K and AKT protein levels, which contrasted with the shNC group. Furthermore, WB and RT‐qPCR manifested a significant underexpression in the INHBA expression across the shCOL10A1 group (Figure 9D,E).

## 4 Discussion

PCa is a prevalent malignancy in males, and recently, prostate‐specific antigen (PSA) testing has significantly raised diagnosis rates [16]. The therapeutic intervention of PCa, especially in instances of metastasis, poses a substantial difficulty due to a restricted comprehension of the molecular pathways that drive cancer advancement [17, 18]. Therefore, comprehending the specific molecular processes underlying the advancement of PCa is imperative for devising improved intervention approaches for individuals diagnosed with the aforementioned condition.

Previous studies have demonstrated that collagen performs a crucial function within TME, function as both a structural scaffold for cellular development and an inducer of epithelial cell proliferation, differentiation and migration [19]. COL10A1, which belongs to the collagen family, has been observed to have significant expression levels in the majority of tumour tissues. Furthermore, its expression has been linked to the process of tumour angiogenesis according to recent research [20]. Previous papers have presented evidence indicating that COL10A1, a gene associated with an adverse prognosis, possesses a crucial involvement in pancreatic cancer progression and metastasis through its involvement in regulating collagen deposition [10]. Meanwhile, it has been documented in separate studies that tumour tissue has elevated COL10A1 gene expression contrasted with corresponding normal tissue in colorectal, lung and oral cancer cases [9, 21, 22]. Nevertheless, COL10A1 biological implication in PCa and its underlying processes remain unknown. Throughout this investigation, utilising data obtained from the TCGA and GEO databases, we determined a significantly elevated expression level of COL10A1 within PCa tissues compared to neighbouring noncancerous tissues, which was further validated using clinical samples. Additionally, heightened COL10A1 expression in PCa patients exhibited significant correlations with T, N and M stages, PSA levels, Gleason score and poorer PFI. The bioinformatic analysis data suggested COL10A1 contributed to PCa progression, prompting further investigation using PC3 cells as an experimental model in vitro. The findings manifested that COL10A1 upregulation facilitated PCa cell progression, migration and invasion while inhibiting apoptosis, whereas downregulation of COL10A1 exerted the opposite effects.

The INHBA gene, which belongs to the transforming growth factor‐β superfamily, is situated on chromosome 7p14.1. It is responsible for encoding the βA‐subunit of activin/inhibin [23]. Over the past few decades, there has been a gradual investigation into the involvement of INHBA in different malignant tumours. The association between INHBA's participation in cancer and activin A levels has been found in the oesophagus, prostate and ovarian malignancies [24, 25, 26]. Researchers manifested that INHBA expression was strongly connected with the diameter and invasion of the tumour within gastric malignancy as a sign of poor prognosis [27]. Multiple investigations have provided evidence indicating that INHBA exerts regulatory effects on cellular growth and apoptosis through the PI3K/AKT pathway [28]. To investigate the underlying molecular processes of COL10A1‐mediated PCa progression further, we discovered that COL10A1 interacts with INHBA and that COL10A1 is positively linked with INHBA in PCa. Moreover, the inhibition of INHBA restored the upregulation of cell progression, migration and invasion stimulated via COL10A1 overexpression, while the overexpression of INHBA counteracted the downregulation of cell progression, migration and invasion caused by suppressing COL10A1.

**FIGURE 10:**
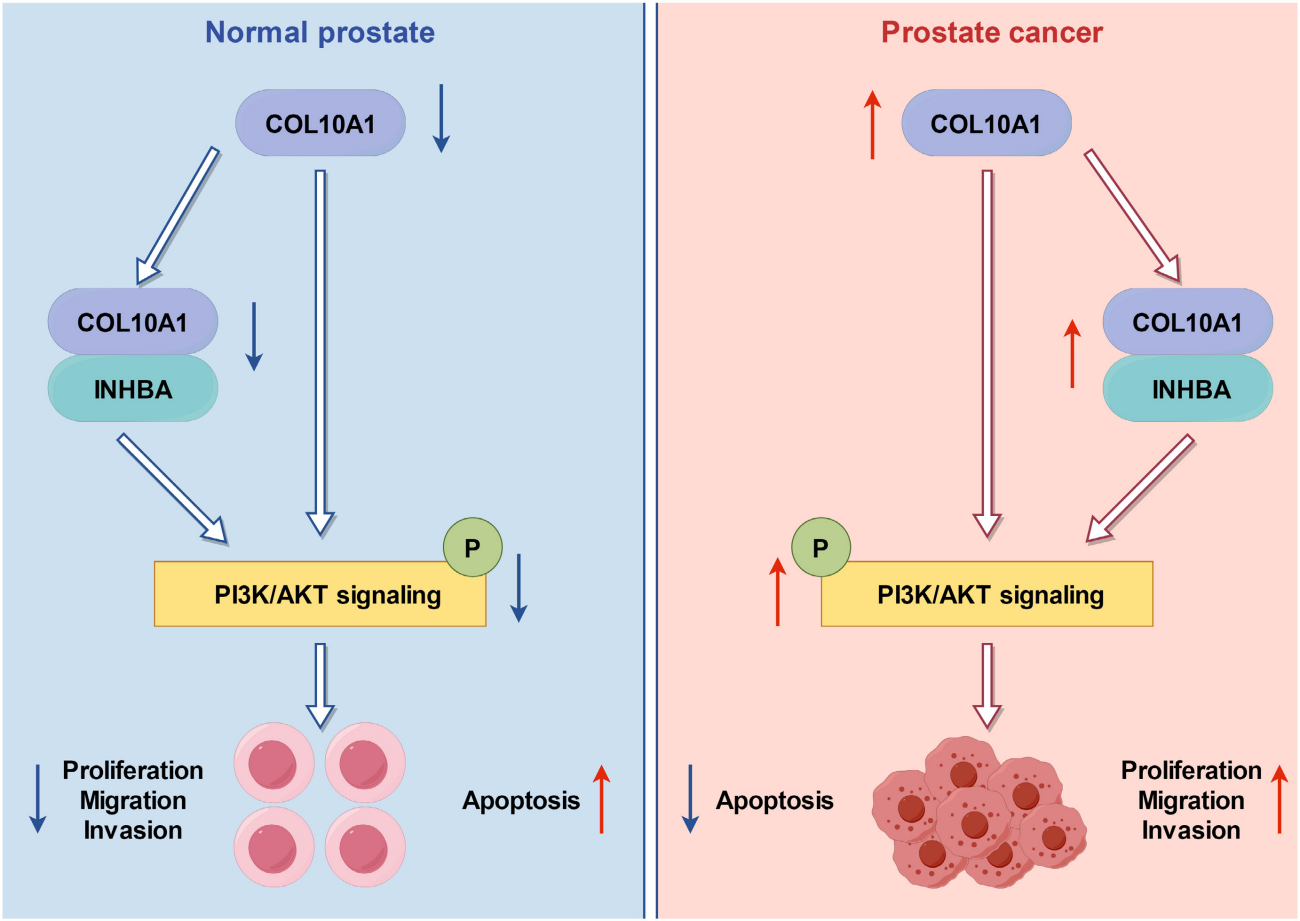
COL10A1 promotes PCa cell progression, migration and invasion By interacting with INHBA.

Metastasis, a hallmark of cancer, is a primary contributor to the failure of cancer therapy and elevated death rates [29]. The complex mechanisms behind cancer metastasis encompass a multitude of signalling pathways [30]. The PI3K/AKT signalling pathway is frequently activated in cancer, with mounting evidence suggesting its association with tumour progression, invasion, metastasis and autophagy [31, 32]. Here, we conducted GO, KEGG and GSEA assays revealing that COL10A1 expression levels are positively related to the PI3K/AKT pathway. Hence, we conducted additional investigations into the protein expression profiles linked to the PI3K/AKT pathway. The results obtained from WB analysis demonstrated that the elevated COL10A1 expression led to a rise in PI3K and AKT phosphorylation levels, which was reversed upon suppressing INHBA. In contrast, the INHBA upregulation avoided the p‐PI3K and p‐AKT downregulation stimulated via COL10A1 silencing. Collectively, our findings demonstrated that COL10A1 activated the PI3K/AKT pathway by interacting with INHBA in PCa Figure 10.

## 5 Conclusion

Collectively, the present study outcomes confirm that COL10A1 performs a significant function in promoting tumour development of PCa by enhancing progression, migration and invasion, besides suppressing apoptosis by the PI3K/AKT pathway activation. This activation is caused by the interaction between COL10A1 and INHBA, both in vivo and in vitro. Accordingly, COL10A1 can be a predictive factor and a promising therapeutic approach for PCa.

## Author Contributions

**Kun Jiang:** investigation (lead), resources (lead), writing – original draft (lead). **Li‐zhe Xu:** data curation (lead), formal analysis (lead), software (lead), visualization (lead). **Fan Cheng:** funding acquisition (lead), methodology (lead), supervision (lead), writing – review and editing (supporting). **Jin‐zhuo Ning:** conceptualization (lead), project administration (lead), writing – review and editing (lead).

## Ethics Statement

All procedures were done according to protocols approved by the Clinical Research Ethics Committees of Renmin Hospital of Wuhan University and conducted in accordance with the guidelines of ethical management. Committee approval number: WDRY2021‐KS010.

## Consent

Written informed consent for publication was obtained from all participants.

## Conflicts of Interest

The authors declare no conflicts of interest.

## Data Availability

The data used and analysed during the current study are available from the corresponding author on reasonable request.
